# Root system architecture for abiotic stress tolerance in potato: Lessons from plants

**DOI:** 10.3389/fpls.2022.926214

**Published:** 2022-09-23

**Authors:** Rasna Zinta, Jagesh Kumar Tiwari, Tanuja Buckseth, Kanika Thakur, Umesh Goutam, Devendra Kumar, Clarissa Challam, Nisha Bhatia, Anuj K. Poonia, Sharmistha Naik, Rajesh K. Singh, Ajay K. Thakur, Dalamu Dalamu, Satish K. Luthra, Vinod Kumar, Manoj Kumar

**Affiliations:** ^1^Indian Council of Agricultural Research (ICAR)-Central Potato Research Institute, Shimla, Himachal Pradesh, India; ^2^Lovely Professional University, Phagwada, Punjab, India; ^3^Indian Council of Agricultural Research (ICAR)-Central Potato Research Institute, Regional Station, Meerut, India; ^4^Indian Council of Agricultural Research (ICAR)-Central Potato Research Institute, Regional Station, Shillong, India; ^5^School of Biotechnology, Shoolini University, Solan, Himachal Pradesh, India; ^6^Indian Council of Agricultural Research (ICAR)-National Research Centre for Grapes, Pune, Maharashtra, India

**Keywords:** abiotic stress, root system architecture, potato, plant, nitrogen, heat, drought

## Abstract

The root is an important plant organ, which uptakes nutrients and water from the soil, and provides anchorage for the plant. Abiotic stresses like heat, drought, nutrients, salinity, and cold are the major problems of potato cultivation. Substantial research advances have been achieved in cereals and model plants on root system architecture (RSA), and so root ideotype (e.g., maize) have been developed for efficient nutrient capture to enhance nutrient use efficiency along with genes regulating root architecture in plants. However, limited work is available on potatoes, with a few illustrations on root morphology in drought and nitrogen stress. The role of root architecture in potatoes has been investigated to some extent under heat, drought, and nitrogen stresses. Hence, this mini-review aims to update knowledge and prospects of strengthening RSA research by applying multi-disciplinary physiological, biochemical, and molecular approaches to abiotic stress tolerance to potatoes with lessons learned from model plants, cereals, and other plants.

## Introduction

The root is an integral plant part that provides anchorage, water, and nutrients to the plant. The aboveground plant parts (e.g., stems and leaves) have been widely investigated by several groups worldwide in crops for abiotic stresses like heat and drought tolerance (Tracy et al., 2020; van der Bom et al., 2020), but limited reports are available on underground roots. The root system architecture (RSA) is the 3-dimensional structure of the root system of a plant in soil, and it is highly essential for efficient water and nutrient acquisition and plant growth and development (Lynch, 2019). Although, substantial information has been generated and deployed in cereals for enhancing nutrient use efficiency (Lynch, 2019, 2021) and some RSA reports are also available on potatoes on heat, drought, and nitrogen use efficiency (Villordon et al., 2014; Tiwari et al., 2018, 2020). Hence, there is an urgent need to enhance knowledge of root architecture in the potato by applying multi-disciplinary physiological, biochemical, and molecular approaches. Thus, RSA profiling is now a promising breeding strategy for developing resource-efficient potato cultivars.

The availability of information on RSA is crucial for abiotic stress tolerance in potatoes, particularly heat and drought stress. In potatoes, the tuber is the most economically important plant part and the root is a vital organ, that supplies nutrients and water for stolon formation, and then tuber growth and development (Iwama, 2008). Considerable research on RSA has been evidenced in cereals (Lynch, 2021) but very little on potatoes (Wishart et al., 2013). For example, maize root ideotype *Steep, Cheap, and Deep* has been designed to increase the efficient uptake of water and nutrients through roots from deep and shallow soils (Lynch, 2019). Likewise, a large number of studies have been illustrated on heat and drought stress tolerance but limited research on the exploitation of RSA for crop improvement, except a few demonstrated toward improving nitrogen use efficiency of plants (van Bueren and Struik, 2017). Genetic variability has been explored in potatoes, including root traits of *N* stress in potatoes (Zebarth et al., 2008; Trehan and Singh, 2013; Ospina et al., 2014; van Bueren and Struik, 2017). Aeroponics technology has also been proven in potatoes for precision phenotyping of root traits for improving *N* use efficiency (Tiwari et al., 2018, 2020). Moreover, exploration of root traits is essential to meet sustainable potato production by improving nutrient and water use efficiency (Garnett et al., 2009; Duque and Villordon, 2019; White, 2019; Tracy et al., 2020). Overall, the focus has been driven to harness the potential of root traits toward increasing nutrient acquisition along with water for abiotic stress tolerance in plants.

## Abiotic stresses in potato

### Heat and drought stress

Potato is considered a crop of cool and temperate climate, and high temperatures inhibit tuber growth and yield due to heat stress. In general, tuberization is reduced at high night temperatures above 20°C with complete inhibition of tuberization above 25°C. Exposure of potato plants to high temperatures alters the hormonal balance in the plants. The heat stress tolerance breeding program considers tuberization under high night temperatures (>22°C). Potato is mostly an irrigated crop in plains, and rain-fed crops in hilly regions. Drought is an emerging problem in potato production due to erratic rainfall and the unavailability of irrigation water (Monneveux et al., 2013). The potato plant is highly sensitive to moisture availability and the decline in photosynthesis is fast and substantial even at relatively low water potentials of −3 to −5 bars. Tuber traits such as shape, cracking, dry matter content, and reducing sugars are highly influenced by the availability of soil moisture during the vegetative period.

### Nutrient, salinity, and cold stress

Macro- and micro-nutrients are essential for good vegetative growth, yield, and quality of potatoes. Potato is a shallow-rooted crop and irrigated cultivation is followed on sandy-loam soils in India. Out of the total *N* fertilizer applied, nearly 40–50% is only used by plants (Garnett et al., 2015). The excessive application of *N* fertilizers in potatoes increases production cost and also causes nitrate leaching, and groundwater contamination, and thereby causes environmental pollution (Tiwari et al., 2018, 2020). Salinity is also another problem that could be due to soil salinity or irrigation water. It causes nutritional imbalances, restricts plant growth, and early senescence, and reduces tuber yield, particularly in semi-arid/arid regions. Besides, cold is one of the problems in temperate regions. Temperatures below −2°C can result in partial or complete loss of crops. In temperate zones, freezing injury can occur during the spring season when the crop is at the initial stage of vegetative growth or during autumn when it is near maturity.

## Root system architecture research in plants

### Determining root ideotypes for efficient nutrient uptake/utilization

Harnessing the potential of RSA is now a priority research to develop varieties for abiotic stress tolerance particularly, heat and drought stress. A root ideotype with narrow and deep root systems is ideal for *N* acquisition. The crops having deeper roots are more efficient in *N* acquisition facilitated by steeper root growth angles, fewer axial roots, lesser lateral branching, and anatomical structures (Lynch, 2021). The *Steep, Cheap, and Deep* root ideotype of maize is beneficial for subsoil foraging or capture of *N* and water from deeper soils, which consists of special architectural, anatomical, and physiological features (Lynch, 2013, 2019). Dechorgnat et al. (2018) witnessed a dense maize root ideotype of brace, seminal, and crown roots with mainly increased crown root profile (root length, surface area, and volume). More aerial nodal roots and fewer crown roots increase the uptake of *N* and deep water under dense planting in maize (Zhang et al., 2018). Lynch (2021) pointed out that crop genotypes with reduced metabolic costs of soil exploration can improve water and nutrient acquisition efficiency. Table 1 summarizes some recent work on root architecture studies in potatoes.

**Table 1:** Root system architecture studies on different abiotic stresses in potato.

**Sr. No**.	**Trait/stress**	**Potato genotype**	**Technology used**	**Key findings**	**References**
1.	High-throughput root-trait phenotyping	Root and tuber crops	High-throughput root-trait phenotyping techniques	Discussed new phenotyping methods based on root branching and nutrient capture, and examined root morphology, anatomy, and germplasm screening with enhanced root architecture. Non-invasive *in situ* imaging in the field were advocated such as X-ray computed tomography, laser, nuclear magnetic resonance (NMR), ground penetrating radar (GPR), infrared (IR) imaging, and near-infrared (NIR) imaging alongside a robust database and data analysis pipeline	Villordon et al. (2014), Khan et al. (2016), Duque and Villordon (2019)
2.	Root morphology	IWA1/2/3/4 Norin 1 Konafubuki	Win-RHIZO software	Root mass showed a negative correlation with early tuber bulking, but a positive correlation with shoot mass and final tuber yield	Iwama (2008)
3.	Potato root architecture	Desirée	ImageJ program (http://imagej.nih.gov/ij/)	Described adventitious root (AR) growth and lateral root (LR) branching. Elucidated understanding of origin and nature of AR systems in potato. Results indicate that LR formation in potatoes follows a similar pattern as in model plants, and facilitates its manipulation to improve soil exploitation and yield.	Joshi et al. (2016), Joshi and Ginzberg (2021)
4.	Canopy development and nitrogen use efficiency	189 cultivars	Field phenotyping	Assessed phenotypic variation for NUE traits in potatoes and determined association between NUE and canopy development under high and low *N* input.	Ospina et al. (2014)
5.	Root traits under *N* stress	Kufri Jyoti, Kufri Gaurav	Aeroponics and WinRhizo software	Demonstrated precision phenotyping of potato roots and determined NUE variables in aeroponics under low and high *N* supply.	Tiwari et al. (2020), Tiwari et al. (2022)
6.	Root traits	28 genotypes (Tuberosum and Phureja groups)	Root excavation from field and glass house screening	Root traits variation indicated that final yield was correlated negatively with basal root length, and weakly but positively with total root weight. Phureja genotypes had more numerous basal roots than stolon roots compared to Tuberosum group.	Wishart et al. (2013)
7.	Drought stress	12 genotypes	Destructive field phenotyping and general linear model (GLM)	Applied field phenotyping to identify the useful traits to an environmental stress. Study showed that stolon root traits were associated with drought tolerance in potato and could be used to select genotypes with resilience to drought.	Wishart et al. (2014)
8.	Root traits under drought stress	Tolerant: Gwiazda and Tajfun Sensitive: Oberon and Cekin	Field phenotyping	Established relationship between root system architecture and drought tolerance. Root dry mass decreased under drought stress, and drought-tolerant cultivars developed elongated roots, unlike drought-sensitive cultivars.	Boguszewska-Mańkowska et al. (2020)
9.	Potato morphology under drought stress	Bintje, Dérirée and many lines	WinRhizo and other softwares	Concluded that small canopies increase harvest index and decrease evapotranspiration, whereas open stem-type canopies increase light penetration and shallow but densely rooted cultivars increase water uptake.	Hill et al. (2021)
10.	Root phenotyping model	Fujin, Zaodabai, and Helanshiwu	3D model for potato roots	Developed 3D models of the tuber-root systems based on topological and geometric structures.	Zhao et al. (2020)
11.	Plant architecture	*Solanum tuberosum* subsp. *andigena* 7540	Grafting	A potential graft-transmissible microRNA *miR156*, a phloem mobile signal, plays vital role in plant architecture and tuberization in potato.	Bhogale et al. (2014)
12.	Root architecture	Desiree, Longshu3	Nikon D3000 digital camera imaging	Potato *miR160a/b* plays key roles in root architecture and auxin signaling-related gene expression. Knockdown *miR160* led to a reduction in root length and fresh weight, and an increase in lateral root number.	Yang et al. (2021)

See examples of other crops in Supplementary File S1.

**Figure 1:**
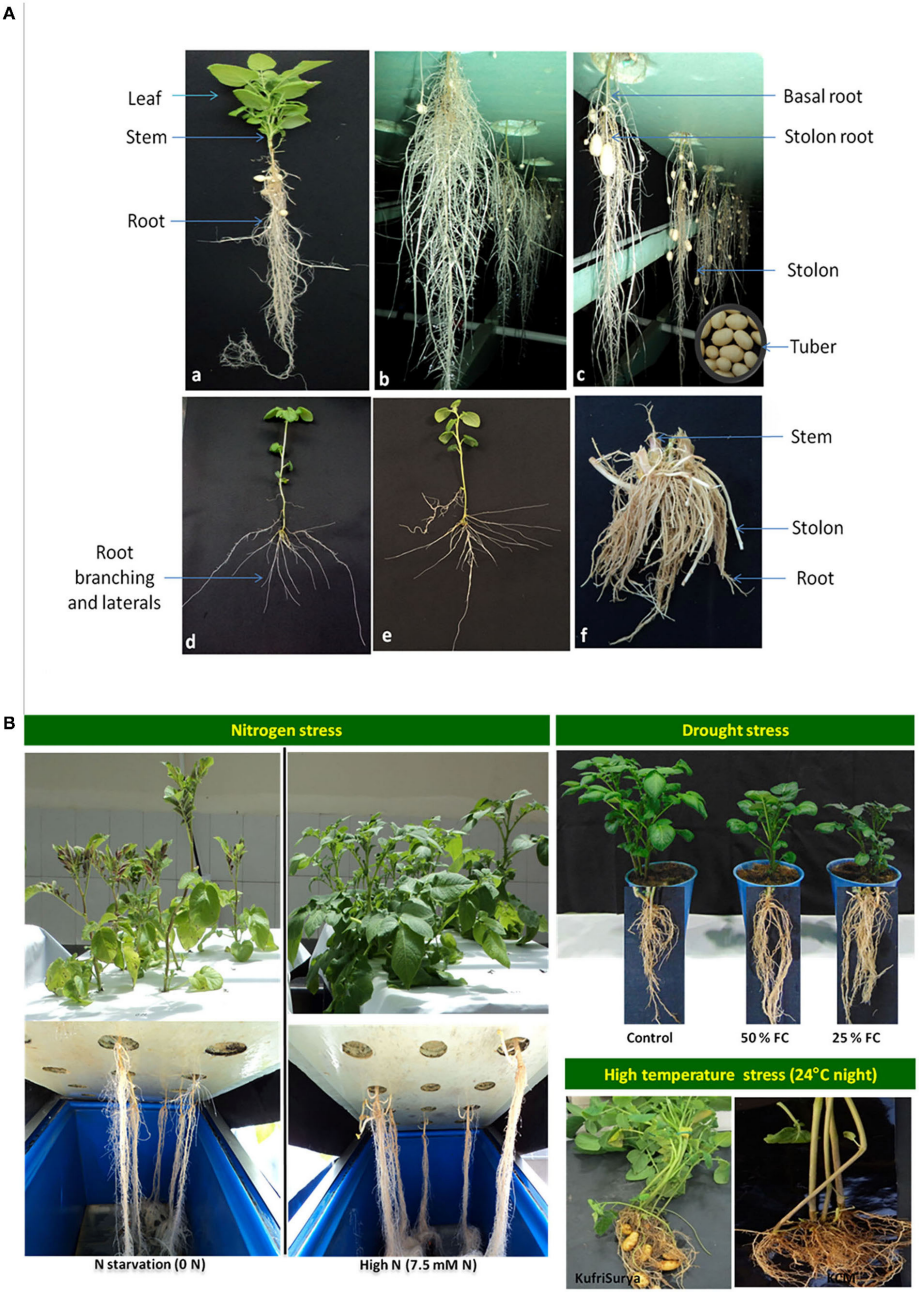
**(A)** Illustrates the root biomass and tuber growth in potato plants grown under aeroponics: a) complete plant growth including root biomass at 45 days after planting (DAP), b) root architecture showing root volume and tuber initiation under hanging roots in aeroponics system at 45 DAP, c) fully grown tubers and harvesting of minitubers at 50 DAP (<5 g each), d, e) pattern of root branching and laterals development at a very early stage (10 days-old seedlings), and f) root biomass of plants at harvesting stage (90 DAP) grown in the field with limited *N* (50 kg/ha) supply. **(B)** depicts plant phenotype (foliage and roots) under different stresses like nitrogen (left) (*N* starvation vs. high *N*), drought (right top) (control, 50% field capacity and 25% field capacity) and high temperature stress (right bottom) (>24°C night temperatures).

The role of the roots-based approach has been described in breeding for nutrient uptake in cereals and other crops (Garnett et al., 2009). It is now clearly evident that root branching plays a very crucial function in nutrient acquisition, which determines plant growth and tuber yield in potatoes (Duque and Villordon, 2019; White, 2019; Tracy et al., 2020). The potato is a model tuber crop species for analyzing underground plant parts like roots, stolons, and tubers (Iwama, 2008). Potato is a shallow-rooted crop that includes basal and stolon roots. The basal roots are involved in plant anchorage and water uptake, whereas the stolon roots capture nutrients and promote tuber growth in potatoes (Villordon et al., 2014). The deeper basal roots and numerous short roots are more advantageous for high tuber yield (White, 2019). It has been determined that basal root length and total root weight are associated with total tuber yield in potatoes (Wishart et al., 2013), and root length and surface area are correlated with higher *N* uptake (Sattelmacher et al., 1990). Khan et al. (2016) suggest that root traits such as root length, spread, number, and length of lateral roots show greater plasticity in response to environmental changes and have better nutrient use efficiency in potatoes. It is evident that the concentration of abscisic acid (ABA) increases in roots under drought stress and shows a linear relationship with stomatal conductance in potatoes (Liu et al., 2005). Drought stress has been witnessed to increase root depth with a high root-to-shoot ratio and allow for uptake of water from deeper strata, whereas, decreased total rot length, increased or decreased root dry mass and stolon number were recorded in different reports (Hill et al., 2021). High-temperature stress reduces plant growth, including reduced root and stolon, delayed tuberization, and thereby lowers tuber yield but increases starch degrading enzymes, heat-sock proteins, and transpiration rate in potatoes (Dahal et al., 2019). Salinity causes reduced root length, root volume, and tuber yield, while it causes stomatal closure and increased ABA, proline, and Na^+^ transport in potato roots (Dahal et al., 2019). Despite these studies, root branching, adventitious roots, and lateral roots, and their mechanism as well as functions in plant growth and development are poorly understood in potatoes. Hence, research on root architecture needs a priority in potato breeding for developing nutrient-use efficient cultivars. In our study, Figure 1A depicts root morphology in potatoes under the field and aeroponics (a soil-less culture of liquid nutrient supply in the mist form) conditions: (a) plant canopy and root biomass at 45 days after planting (DAP) in aeroponics, (b) hanging root growth and tuber initiation in aeroponics system at 45 DAP, (c) harvesting of minitubers at 50 DAP in aeroponics, (d–e) pattern of root branching and laterals development of 10 days-old seedlings in aeroponics, and (f) root biomass of plants at harvesting stage (90 DAP) grown in the field with limited *N* (50 kg/ha) supply. In our other studies, Figure 1B illustrates plant foliage and roots under different stresses like nitrogen (*N* starvation vs. high *N*), drought (control, 50% field capacity soil moisture, and 25% field capacity soil moisture), and high temperatures stress (>24°C night). In aeroponics, we observed variable responses for root traits in 56 potato varieties under optimal *N* supply (Tiwari et al., 2022), and in two contrasting varieties under *N* stress and *N* sufficient conditions (Tiwari et al., 2018, 2020). Under heat stress, lesser root growth and little or no tuber formation were observed at above 24°C night temperatures in potatoes compared to the counterpart, and similar was the case under drought stress. Taken together, the effects of these stresses are detrimental to plant growth particularly root and stolon development, and thus reduces tuber yield in potatoes.

### Multi-disciplinary approaches to investigate root traits

A large number of agronomic, physiological, breeding, and molecular works substantiate root traits studies in many plants such as *Arabidopsis thaliana* (De Pessemier et al., 2013) and tomato (Abenavoli et al., 2016) targeting different traits (Table 1). The significance of RSA to improve nutrient acquisition, translocation, remobilization, and nutrient utilization efficiency has been remarkably known in plants (Chen and Liao, 2017). Recently, Lecarpentier et al. (2021) measured the plasticity of RSA, where lateral root density played a key role under limited *N* in *Brassica napus*. Sinha et al. (2020) documented genetic variation in wheat RSA traits and association with high- and low-affinity nitrate transport systems under optimum and limited *N*. The roles of nitrate and amino acid regulation of shoot branching, flowering, and panicle development, as well as cell division and expansion in shaping of plant architecture have been demonstrated in cereals (Luo et al., 2020). In plants, *N* regulation of root branching (Walch-Liu et al., 2006) and the process of nitrate- and auxin-mediated regulation of root structure have been unveiled recently (Hu et al., 2021).

In potatoes, very high genetic variability has been observed in varieties/germplasm for yield, *N* use efficiency (NUE), and root traits under field conditions (Errebhi et al., 1999; Zebarth et al., 2008; Vos, 2009; Ospina et al., 2014), and aeroponics (Tiwari et al., 2020). Studies confirmed that genetic variation in root traits and root dry weight was positively correlated with final tuber yield (Sattelmacher et al., 1990; Stalham and Allen, 2001; Wishart et al., 2013). Recently, our aeroponics study demonstrated a highly significant and positive correlation between root traits and tuber yield along with *N* use efficiency in potatoes (Tiwari et al., 2022). In RSA studies on drought stress in potatoes, Wishart et al. (2014) evidence that stolon roots are associated with drought tolerance in fields, whereas elongated roots are noticed in drought-tolerant cultivars (Boguszewska-Mańkowska et al., 2020). Recently, Hill et al. (2021) concluded that an open stem-type plant canopy increases light penetration and shallow but densely rooted cultivars increase water uptake under drought stress in potatoes. The adventitious roots of potatoes are formed post-embryonically from consecutive nodes on shoots and also include lateral root formation through auxin-dependent cell cycle activation, whereas the tap/primary root is formed in the embryo (Joshi et al., 2016). Thus, the root elongation, growth angles, lateral branching, and longevity are governed by genetic, physiological, and environmental factors in potatoes (Joshi and Ginzberg, 2021).

The knowledge about genes involved in *N* metabolism is an essential requirement for providing abiotic stress tolerance by applying modern genomics tools. The understanding of the genes/quantitative trait loci (QTLs) regulation of the symbiotic associations between host plants and arbuscular mycorrhiza fungi or rhizobial bacteria is an important strategy to enhance nutrient acquisition (Li et al., 2016). In rice, the QTL *DEEPER ROOTING 1* was evidenced to increase root growth angle to increase *N* uptake under limited *N* availability (Arai-Sanoh et al., 2014). Kiba and Krapp (2016) highlighted increasing nitrogen acquisition efficiency through high-affinity nitrogen transporters, and root architecture modifications through low-nitrogen-availability-specific regulators of primary and lateral root growth under low *N*. Another study illustrated genomic regions for marker-assisted selection on root morphology in *Brassica napus* (Wang et al., 2017). MicroRNAs play key roles in abiotic stress tolerance like plant adaptation under limited *N* (Khan et al., 2011; Fischer et al., 2013). Recently, Shi and Tong (2021) demonstrated that the *TaLAMP1* gene expression determines wheat plant architecture by regulating spike number/plant and grain number/spike in response to *N* (Table 1). Many genes have been proven in various plants such as Arabidopsis for root architecture (e.g., *NRT1.1, TAR2, AHA2*, and *miR393*) and lateral root development (e.g., *NRT2.1, CLE-CLV1*, and *miR167*); and root architecture modification in rice (*OsMADS25, EL5*, and *OsNAR2.1*) and wheat (*TaNAC2-5A*; review by Li et al., 2016). The root-specific *N* transporters such as nitrate transporter (NRT), ammonium transporter (AMT), and signaling molecules or regulatory elements (transcription factors and miRNAs) could be targeted for engineering new genotypes with better nutrient use efficiency, heat and drought stress tolerance. The *miR156* is a potential graft-transmissible and a phloem mobile signal that plays a key role in plant architecture and tuberization (Bhogale et al., 2014). Recently, a study illustrated that *miR160a/b* participates in root architecture and auxin signaling-related gene expression in potatoes. The knockdown *miR160* led to a reduction in root length and fresh weight but an increase in lateral root number (Yang et al., 2021). Overall, integrated agronomic, physiological, breeding, and molecular research strengthens our understanding of root architecture.

## High-throughput phenotyping to dissect root architecture

The available approaches for root phenotyping in laboratory, greenhouse, and field include simple agar plates to labor-intensive root digging (“shovelomics”) and soil boring methods, the construction of underground root observation stations, and now sophisticated computer-assisted root imaging techniques. A wide range of high-throughput phenotyping (HTP) systems has been demonstrated in crop species to measure RSA and multiple plant phenotypic traits (Nguyen and Kant, 2018; Tracy et al., 2020). Table 1 outlines the RSA studies on potatoes. Paez-Garcia et al. (2015) summarized root architectural traits relevant to crop productivity and developed root phenotyping strategies for crop and forage breeding programs. A scanner system has been developed for high-resolution quantification of root growth dynamics in *Brassica rapa* (Adu et al., 2014). Araya et al. (2016) applied a statistical modeling approach to investigate modulations of root architecture in *Arabidopsis thaliana* in response to varied *N* availability. The RhizoTubes system has been deployed for high throughput imaging of plant roots architecture in the model plant *Medicago truncatula*, crops like *Pisum sativum, Brassica napus, Vitis vinifera, Triticum aestivum*, and weed species *Vulpia myuros* (Jeudy et al., 2016). The selection criteria have been developed for spinach roots under low *N* using machine learning tools based on root architecture traits such as the number of root tips, root length, crossings, and root average diameter (Awika et al., 2021). Thus, HTP methods and simulation models will necessarily speed up the trait improvement by non-destructive simultaneous phenotyping of both roots and shoots.

A non-invasive and non-destructive phenotyping technique warrants special attention for a more accurate assessment of root traits in response to various stresses, for instance, limited *N* availability. In recent years, different platforms have been developed for root HTP under environment-controlled as well as natural field conditions, such as the in situ root imaging technique (Richner et al., 2000). Han et al. (2009) successfully applied the X-ray computed tomography (CT) technique to extract the architecture of first-order roots in potatoes. The magnetic resonance imaging (MRI) technique can be deployed to assess RSA in the early stage of potato growth (Monneveux et al., 2013). Root phenotyping techniques comprise some degree of automation with imaging, image analysis, and processing. Various imaging and its analysis techniques/software have been found effective as reliable tools for root phenotyping, such as WinRhizo, Smart Root, EZ-Rhizo, Image J, Root System Analyzer, Root Nav, IJ_Rhizo, and Root Trace (Wasaya et al., 2018). Duque and Villordon (2019) discussed the role of investigation on root morphology and anatomy under *N* stress in potatoes. Recently, 3D models have been developed for tuber-root systems based on topological and geometric structures (Zhao et al., 2020). The advancement in sensor technology allows for measuring root architecture and tuber growth in potatoes. The use of HTP at the harvesting stage will help to assess tuber characteristics such as shape, size, skin color, texture, and number, and to predict yield. Moreover, hyperspectral and multispectral imaging could be used to assess the tuber quality parameters like carbohydrates, starch, protein, reducing sugar, and water content. Thus, there is an immense opportunity to harness the potential of HTP in potato improvement via dissection of root traits.

## Concluding remarks and future perspectives

Nutrients, heat, drought, salinity, and cold are the important abiotic stresses of potatoes. Excessive application of chemical fertilizers, mainly nitrogen, increases production costs and causes a negative impact on the environment. Knowledge of root architecture, anatomy, and function is important for nutrient-efficient crop breeding (Lobet et al., 2019). Root architecture plays a very essential role in plant anchorage, nutrient and water acquisition, and environmental benefits such as carbon sequestration and reducing soil erosion. Unlike advanced research on rice, wheat, and maize, limited information is available on RSA in potatoes. Given that *N* compounds are mobile and prone to leaching underground, a shallow-rooted potato root ideotype can capture nutrients and water from top soils, whereas a deep root ideotype would be advantageous for deeper soils. In addition, the molecular and genetic basis and physiological or developmental regulation of basal and stolon root architecture variation will greatly benefit breeding for abiotic stress tolerance (Kochian, 2016). Information about the association between basal and stolon roots architecture vis-a-vis carbon partitioning and tuber yield remains unclear in potatoes. The advancement in modern technologies such as sensors, robotics, cameras, and HTP platforms allow dissection of root architecture and phenomics-based crop breeding. Concomitantly, it is a challenging task due to massive data and computation analysis. In the 21st century, crop breeding will shift from single root traits to rhizosphere selection and phenotype-based crop improvement. Profiling RSA and its application is a promising and underexploited avenue to address climate-resilient and resource-efficient crops that are urgently needed in global agriculture. Thus, a renewed emphasis is needed on root architecture to develop abiotic stress-tolerant varieties by applying modern genomics tools.

## Author contributions

JT conceived the idea and wrote the manuscript. All authors contributed to the research work and literature, editing, and approving the manuscript for publication.

## Funding

This work is funded under the institute Biotechnology program by ICAR-CPRI, Shimla, CABin Scheme, and ICAR-LBS Outstanding Young Scientist Award project to JT.

## Conflict of interest

The authors declare that the research was conducted in the absence of any commercial or financial relationships that could be construed as a potential conflict of interest.

## Publisher's note

All claims expressed in this article are solely those of the authors and do not necessarily represent those of their affiliated organizations, or those of the publisher, the editors and the reviewers. Any product that may be evaluated in this article, or claim that may be made by its manufacturer, is not guaranteed or endorsed by the publisher.
