# Supplementary material for: Root system architecture for abiotic stress tolerance in potato: Lessons from plants
Source: Front Plant Sci. 2022 Sep 23;13:926214. doi: 10.3389/fpls.2022.926214 (PMC9539750; doi:10.3389/fpls.2022.926214)
Supplement: Supplementary file 1 [file Table_1.DOCX]

**Supplementary File S1:** Selected root system architecture research in other plants

| Tomato | Measured NUE and root traits such as root length, surface area, and root volume, shoot and root dry weights, root length ratio, root mass ratio, root thickness or fineness and root density. | Abenavoli et al., 2016 |
| --- | --- | --- |
| *Arabidopsis thaliana* | The variation of the root biomass and RSA traits like primary root length and lateral roots (number, length and densities) help to develop plants with a dense and profound root system for higher N uptake efficiency. | De Pessemier et al., 2013 |
| *Arabidopsis thaliana* | Developed a statistical modeling approach to investigate modulations of RSA in response to nitrogen availability. | Araya et al., 2016 |
| Maize | Root ideotype (steep, cheap and deep) is applicable to low-input systems because of deep rooting for water and N acquisition from deeper soil strata. | Lynch, 2013 |
| Maize | The root ideotype of genotype B73 presented a higher density root system (brace, seminal, and crown roots per plant) than the roots of F44, which increased its crown root profile (length, surface area and volume). | Dechorgnat et al., 2018 |
| Maize | More aerial nodal roots and fewer crown roots favored in temperate maize for root-lodging resistance and uptake of deep water and nitrogen under dense planting. | Zhang et al., 2018 |
| Wheat | Gene *TaLAMP1* expression determined plant architecture by regulating spike number/plant and grain number/spike in response to N. | Shi and Tong, 2021 |
| Wheat | Identified genetic variation in RSA traits and association with high- and low-affinity nitrate transport systems under optimum and limited N. | Sinha et al., 2020 |
| *Brassica rapa* | Developed a scanner system for high-resolution quantification in root growth dynamics. | Adu et al., 2014 |
| *Brassica napus* | Study provided genomic regions for MAS on root morphology based NUE improvement. | Wang et al., 2017 |
| *Brassica napus* | Measured plasticity of RSA, where lateral root density played key roles in under limited nitrogen. | Lecarpentier et al., 2021 |
| Spinach | Developed selection criteria based on root architecture using machine learning tools. Number of root tips and root length under high N, and crossings and root average diameter under low N were relevant traits. | Awika et al., 2021 |
| Model, crop and weed plants | Developed RhizoTubes system for high throughput imaging of plant roots architecture in model plant *Medicago truncatula,* crops like *Pisum sativum, Brassica napus, Vitis vinifera, Triticum aestivum*, and weed *Vulpia myuros*. | Jeudy et al., 2016 |
| Cereals | Discussed role of nitrate and amino acid regulation of shoot branching, flowering, and panicle development, as well as cell division and expansion in shaping plant architecture. | Luo et al., 2020 |
| Plants | Root based approaches to improving NUE in plants through N acquisition and assimilation through root morphology, root to shoot ratio, root vigour, root length density, and root N transport and metabolism. | Garnett et al., 2009 |
| Plants | Discussed role of auxin and process of nitrate- and auxin-mediated regulation of root structure. | Hu et al., 2021 |
